# Challenges to timely identify and treat anorexia in aging in the context of the Integrated Care for Older People (ICOPE) Program

**DOI:** 10.1016/j.jarlif.2025.100035

**Published:** 2025-12-31

**Authors:** Kelly Virecoulon Giudici

**Affiliations:** Institute of Aging, Gerontopole of Toulouse, Toulouse University Hospital, Université Toulouse III Paul Sabatier, Toulouse, France

**Keywords:** Appetite, Anorexia of aging, Nutritional status, Intrinsic capacity, Longevity, Malnutrition, Frailty, Sarcopenia

## Abstract

Frailty and sarcopenia represent harm to good longevity and are often related to inadequate dietary intake and to a decrease in appetite over the years, which are characteristics of a complex process also known as anorexia in aging. Understanding the factors leading to anorexia in aging is crucial for enabling the adequate development of public policies and clinical strategies to prevent and treat this condition and to help older adults to pursue healthy aging pathways. In the present article, a brief overview on the factors contributing to appetite loss and malnutrition among older adults is presented, and challenges to timely identifying and treating anorexia in aging are discussed. Major factors known to affect appetite and favor lower food intake in older people include physiological, pathological or social conditions. Trials testing treatments for anorexia in aging have focused on education, exercise, meal adjustments, nutritional supplementation and medications, but results are variable, partly due to the multitude of etiological factors and determinants of appetite loss in older adults, demanding further research. Routine screening in primary care with simple tools, as the Integrated Care for Older People (ICOPE) program, might important contribute for preserving intrinsic capacity and nutritional status, as also to early identifying the need for treating anorexia in aging.

## 1 Introduction

Frailty and sarcopenia represent harm to good longevity and are often related to inadequate dietary intake and to a decrease in appetite over the years, which are characteristics of a geriatric syndrome known as anorexia in aging [[1], [2], [3]]. Anorexia in aging has been associated with increased mortality risk, not only among hospital inpatients and long-term care residents, but also among community-dwelling older adults [[4], [5], [6]]. Moreover, when such condition is combined with weight loss, risk is even higher [7]. Studies estimate that the prevalence of anorexia in aging varies from 25 % to 40 % [8,9]. Nevertheless, this condition is believed to be under-diagnosed [5,9,10], which means that its impact on public health might be even higher.

As appetite is a major determinant of nutritional status [4], it is probable that undiagnosed and untreated anorexia in aging may evolve to weight loss and undernutrition, negatively impacting quality of life and favoring strength decrease, loss of autonomy and care dependency [2,[11], [12], [13]]. However, a remarkable challenge in timely identifying and adequately treating anorexia in aging lies in the fact that it can be triggered by a wide variety of determinants [1,11,14].

Understanding the factors leading to anorexia in aging is therefore crucial for enabling the adequate development of public policies and clinical strategies to prevent and treat this condition and to help older adults to pursue healthy aging pathways. In the present article, a brief overview on the factors contributing to appetite loss and malnutrition among older adults is presented, and challenges to timely identifying and treating anorexia in aging are discussed.

## 2 Why does appetite tend to decrease in later adulthood?

Considering the physiological aspects determining dietary intake, alterations in the oral cavity, olfactory apparatus and in gastrointestinal function are frequent among older adults [14,15]. In the mouth, we may have teeth loss, mouth dryness and reduced quantity and sensitivity of lingual papillae (leading to reduced capacity of tasting food) affecting appetite [15]. Decrease in olfactory cells impairs the ability to adequately smell food as well [15]. Swallowing difficulties, alterations in gastric motility and delayed gastric emptying are also often observed [14,16].

Difficulties in performing instrumental activities of daily living (IADL) is associated with anorexia in aging [17]. Diseases affecting IADL (as stoke, arthritis and Parkinson’s disease) may trigger the need for assistance in erstwhile simple activities such as shopping food and cooking, being one way by which decreased appetite and dietary intake occur [14]. Moreover, varied biological pathways might explain how pathological factors such as depression, congestive heart failure (CHF), chronic obstructive pulmonary disease (COPD), dementia and cancer can also drive appetite loss [1,14]. The use of medications may also have lack of appetite as a side effect [1,14,18].

Furthermore, anorexia in aging may also be driven by social determinants as loneliness, anxiety, lack of cooking skills, food insecurity and limited income [1,10,14].

As these multiple determinants may affect older adults simultaneously, the more conditions they present, the more susceptible to present restricted energy intake and nutrient deficiency they will be.

## 3 Diagnosing anorexia in aging and challenges to restore good appetite

In the given context, public policies and clinical care procedures aiming to preserve (or restore) good appetite over the aging process must focus on controlling its main causes, as also to find adequate treatments when the problem is already installed.

With nutritional status being recognized as a core measure in the Integrated Care for Older People (ICOPE) program developed by the World Health Organization (WHO) [19], it is crucial to early identify at-risk older adult populations in primary care. The ICOPE was designed to easily screen and assess deficits in intrinsic capacity (IC), and to direct patients in need to the adequate treatment aiming to preserve functional abilities for as long as possible [19]. In ICOPE, nutritional status is considered the basis of the vitality domain in the IC concept, whose initial screening (step 1) is done by asking the patient if he/she have unintentionally lost >3 kg over the last three months, and if he/she have experienced loss of appetite [19]. For those answering yes, the Mini Nutritional Assessment (MNA) [20] should be then applied (step 2) [19], enabling a more in-depth evaluation – among other issues related to malnutrition – of anorexia in aging. Adopting the ICOPE in primary care is therefore a good way to guarantee that appetite loss and anorexia in aging will not be underdiagnosed and untreated.

In addition to the MNA, several other tools might be used in research and clinical care to assess anorexia in aging. A recent scoping review identified 30 different types of appetite assessment methods used in older adults living in community settings [21]. Besides exploring unintentional weight loss and self-perception of appetite decrease, other important topics to evaluate anorexia in aging that are investigated in such instruments include asking the patient about: chewing or swallowing difficulties; ability to eat without assistance; number of meals per day; food taste perception; mood; the use of supplemental drinks and enteral nutrition (tube feeding) [20,21]. In spite of lack of consensus on the best tool for this population, Likert-scales – particularly the Simplified Nutritional Assessment Questionnaire (SNAQ), the Hunger and Sensory Perception Questionnaire (AHSPQ), and the Council on Nutrition Appetite Questionnaire (CNAQ) – were the more frequently used methods [21]. From them, SNAQ stood out for its good validity and reliability, and for being translated into various languages [21]. Moreover, its applicability has been further expanded with the development of a telephone-administered version (T-SNAQ) [22] and with its integration into the Rapid Geriatric Assessment (RGA) [23].

The low cost and the simple applicability of screening instruments majorly based on self-reported changes, like the ICOPE, stand out as advantages in the identification of anorexia in aging. However, with the advance of technologies, and when more financial and human resources are available, expanding the efforts for better capturing signals related to appetite decline in adults and older adults could enable earlier interventions. This might be done by combining these validated tools with additional objective assessments, as anthropometric measures and blood biomarkers. Studies aiming at identifying biomarkers of anorexia in aging have explored anorexigenic and orexigenic hormones, inflammatory markers and DNA methylation-based epigenetic clocks; results are still preliminary and further research is necessary though [24,25].

As multiple conditions may lead to anorexia in aging, potential treatments are also numerous. From orexigenic drugs to social integration activities, several pathways may help re-establishing appetite and increasing food intake [9,15,[26], [27], [28]]. In a systematic review exploring the effectiveness of interventions for anorexia in aging, Cox et al. [26] selected 18 studies which tested nine different intervention types (or their combination) from five categories: education (nutritional counseling), exercise (exercise program), meal adjustments (flavor enhancement; increased meal variety; mealtime assistance), supplementation (fortified food; oral nutritional supplement; amino acid precursor), and medication (megestrol acetate, an appetite stimulant; nandrolone decanoate, an anabolic steroid). Only five of them (flavor enhancement, the three supplementation types and megestrol acetate medication) showed positive effects on appetite when compared to controls or to baseline, but authors called attention to the limited generalization of the findings and to a lack of clarity about whether anorexia in aging or undernutrition was the target of interventions [26].

In addition to the two medications mentioned above, other pharmacological treatments targeting anorexia in aging include peristalsis- and appetite-stimulating drugs, corticosteroids and anabolic steroids, but involve risks of adverse events and interactions with other medicaments in use, so their indication and prescription must be carefully considered [9].

The observed effects of actions related to food consumption in treating anorexia in aging, although limited, call attention to the potential role of food industry on contributing to the fight against this condition. Considering all the mentioned causes of appetite reduction that affect older adults, producers clearly face the opportunity to develop more appealing and palatable foods that are, at the same time, nutritionally adequate, with improved digestibility and higher bio-accessibility of nutrients. In this sense, strategies could include the use of visually attractive vibrant colors (obtained with food colorings of natural origin), diverse textures (soft but not mushy, achieved with technologies such as gelation, enzyme treatment and blade tenderization), and stronger flavors based on non-salty herbs, spices and umami-rich ingredients (as mushrooms, tomatoes and nutritional yeast) [29].

Among the lifestyle interventions, actions promoting social integration and fighting sedentary behavior seem to have a potential in treating anorexia in aging [27,28]. Inspired by a connection observed between physical activity and increased appetite in young adulthood, Turesson et al. [28] conducted a review exploring the potential link between these conditions among older adults. Although a considerable variation in research design and methodologies, such association was observed among this older population in most part of the 25 included studies. Physical activity is believed to contribute to affect appetite not only through physiological effects on appetite-regulating hormones, gastric emptying speed [30], but also by improving mood [31] and fighting loneliness [32], thus acting as a psychosocial stimulator.

These interesting findings and their relevant limitations shed light on the efforts needed to identify the best lifestyle interventions and safe pharmacological treatments able to improve appetite and protect from weight loss (especially muscle mass). Designing future clinical randomized controlled trials (RCT) should ideally take into consideration the multitude of etiological factors and determinants of appetite loss when recruiting participants. However, it is comprehensible that subdividing anorexia in aging into various subtypes would increase complexity of study protocols, perhaps even making it unfeasible to achieving sample sizes with adequate statistical power. A simpler possibility would be to limit study inclusion to one of the three main determinant spheres of anorexia in aging (physiological, pathological or social), and to propose interventions in accordance to that; or, as highlighted in the recent International Conference on Frailty and Sarcopenia Research (ICFSR) Task Force Report [10], to target subjects presenting either anorexia associated with cachexia, or those with anorexia and no cachexia.

Finally, regardless of the type of treatment, it is important to acknowledge that increasing appetite in subjects with anorexia in aging may not be enough to promote adequate nourishing. Nutritional advice should thus be part of the set of assistance measures employed by health professionals to guarantee healthy and adequate dietary intake and to avoid micronutrient deficiencies and/or the development or aggravation of diet-related chronic diseases, such as type 2 diabetes, hypertension and osteoporosis.

## 4 Final considerations

World population is currently getting older in two different main nutritional pathways: whereas we have an increasing prevalence of overweight and obesity [33], frailty, sarcopenia and undernutrition are also important issues to health systems [34]. More deeply, we have the intersection of both – being overweight does not mean being well nourished. Obesity itself has been frequently associated with poor nutritional status, low strength, micronutrients deficiencies and nutrition-related chronic diseases [33]. Furthermore, complex conditions as osteosarcopenic obesity are increasing [35]. In this scenario, anorexia in aging is a geriatric syndrome that may affect older adults regardless of their body mass index (BMI), being an important risk factor to developing malnutrition and, in consequence, leading to reduced strength, loss of autonomy and care dependency [2,[11], [12], [13]].

In the present article, factors related to the decrease of appetite over lifetime were explored, and given the variety of possible causes (which may be unique, or a combination of physiological, pathological or social conditions), challenges to identify and fight this concerning trend were discussed. Screening for unintentional weight loss and decrease in appetite in primary care, as proposed by the ICOPE program developed by the WHO, is a simple and inexpensive way of timely identifying at-risk older adults and driving adequate treatments to them.

Pharmacological treatments involve potential adverse events and should be carefully considered. On the other hand, oral nutritional supplementation is safer and have shown positive results in some studies [26]. Other lifestyle interventions, such as promoting social integration and stimulating physical activity, may be helpful when anorexia in aging is related to depression, loneliness and sedentary behavior.

Future research on the topic face the challenge of finding effective treatments to each cause of anorexia in aging. Limiting study inclusion to one of the three main determinant spheres of anorexia in aging (physiological, pathological or social), or targeting subjects presenting either anorexia associated with cachexia, or those with anorexia but no cachexia may be options for optimizing participants’ recruitment in future trials.

Overall, health care routines from early adulthood targeting on preserving IC and including nutritional advice might contribute to protecting appetite and adequate food intake, and to promoting quality of life and a healthy aging process. Moreover, adopting inexpensive and simple screening tools in care systems may reduce under-diagnosis of anorexia in aging and favor early treatments, minimizing associated comorbidities and related health care burden. As reduced appetite could be interpreted as an early signal for performance decline and resilience impairment, this is particularly interesting in the context of the Brain Watch framework, an innovative concept that integrates cognitive, behavioral and functional performance with traditional physiological metrics aiming to sustain brain performance and wellness [36].

## CRediT authorship contribution statement

**Kelly Virecoulon Giudici:** Writing – original draft, Conceptualization.

## Declaration of competing interest

The authors declare that they have no known competing financial interests or personal relationships that could have appeared to influence the work reported in this paper.
